# Soluble B-cell maturation antigen as a serum marker of MRD in patients with multiple myeloma

**DOI:** 10.3389/fonc.2025.1631511

**Published:** 2025-09-09

**Authors:** Erin A. Dean, Derek M. Li, Tuo Lin, Edith M. Sampson, Robert P. Seifert, Jack W. Hsu, John W. Hiemenz, John R. Wingard

**Affiliations:** ^1^ Department of Medicine, Division of Hematology/Oncology, University of California, Irvine, Orange, CA, United States; ^2^ University of Florida (UF) Health Cancer Center, University of Florida, Gainesville, FL, United States; ^3^ Department of Biostatistics, University of Florida, Gainesville, FL, United States; ^4^ University of Florida (UF) Interdisciplinary Center for Biotechnology Research (ICBR), Monoclonal Antibody Core, University of Florida, Gainesville, FL, United States; ^5^ Department of Pathology, University of Florida, Gainesville, FL, United States; ^6^ Department of Medicine, Division of Hematology and Oncology, University of Florida, Gainesville, FL, United States

**Keywords:** multiple myeloma, MRD, sBCMA, autologous HSCT, biomarker

## Abstract

**Introduction:**

Serum soluble B-cell maturation antigen (sBCMA) has been shown to correspond to high disease burden in uncontrolled Multiple Myeloma (MM). However, it has not been extensively evaluated as a biomarker of minimal/measurable residual disease (MRD).

**Methods:**

In this prospective observational correlative study, the primary objective was to correlate serum sBCMA with tumor burden in the bone marrow (BM) of patients with MM evaluated for first or salvage autologous stem cell transplantation. Paired samples were collected from 44 patients. BM overt disease was identified on morphological analysis or by standard flow cytometry (limit of detection (LOD) of 10-1). BM MRD was assessed by MRD flow cytometry (sensitivity of 1 aberrant clonal plasma cell in 10^5^ nucleated cells) and/or next-generation sequencing (LOD of 10-6).

**Results:**

For transplant recipients (n= 36), the mean serum sBCMA (standard deviation (SD)) was 18.1 (11.7) ng/mL, BM overt disease was present in 12 (33.3%) patients and MRD only/No MRD in 20 (55.6%) patients. For non-transplanted patients (n= 8), the mean serum sBCMA was 9.7 (5.2) ng/mL, BM overt disease was present in 1 (12.5%) patient, while MRD only/No MRD in 5 (62.5%) patients. Serum sBCMA was associated with overt disease (p < 0.001), as well as MRD only/No MRD (p= 0.002). On multivariable logistic regressions modeling, higher serum sBCMA indicated higher odds of BM overt disease (odds ratio (OR) = 1.12, p = 0.007) and lower odds of MRD only/No MRD (OR = 0.91, p = 0.03).

**Conclusion:**

Serum sBCMA was associated not only with BM overt disease, but also with BM detectable or below LOD MRD.

## Introduction

Multiple Myeloma (MM) is a rare, incurable hematologic malignancy, which requires close monitoring of disease status for optimal ongoing management. The depth of response to treatment, especially the absence of detectable minimal/measurable residual disease (MRD) in the bone marrow (BM), has been associated with significant improvement in progression free survival and overall survival in transplant-eligible and ineligible patients with either newly diagnosed or relapsed/refractory (R/R) MM (1). However, serial sampling of the BM is not practical as a biomarker for MRD.

Non-invasive biomarkers for peripheral MRD detection in MM are under investigation, including circulating tumor DNA (ctDNA) derived by next generation sequencing (NGS) and circulating tumor plasma cells identified by multiparametric flow cytometry (2). Thus far, ctDNA has been found to be of limited value as demonstrated in one study where 61% had undetectable peripheral MRD despite having less than very good partial response to treatment (3), and in another study, where 69% of patients had undetectable peripheral MRD despite MRD being detected by NGS in the BM (4).

When cleaved by the enzyme γ-secretase, B-cell maturation antigen (sBCMA), a marker on the surface of B cells essential for the survival of plasma cells, becomes soluble in the bone marrow and periphery (5). Serum sBCMA levels have been shown to correlate strongly with sBCMA from supernatants of cultured BM mononuclear cells in active MM (6). Importantly, higher serum sBCMA levels correspond to higher disease burden in patients with untreated MM compared to those with monoclonal gammopathy of undetermined significance and healthy individuals, as well as higher disease burden in patients with progressive disease versus (vs.) responders (6). While serum sBCMA has been noted to correlate with overt disease activity, it has not been evaluated extensively as a marker of MRD.

In this prospective observational correlative study, we assessed paired peripheral blood and BM specimens in patients with MM pre-autologous hematopoietic stem cell transplantation (HSCT) to determine the relationship between serum sBCMA and BM MRD, as well as serum sBCMA and serum standard myeloma markers. In patients who proceeded to HSCT, we also monitored serum sBCMA with serum standard myeloma markers for up to +12 months post HSCT. By building a prediction model, we investigated the ability of serum sBCMA to predict BM plasma cell neoplasm burden at the time of pre-HSCT.

## Materials and methods

### Patients and study design

**Figure 1:**
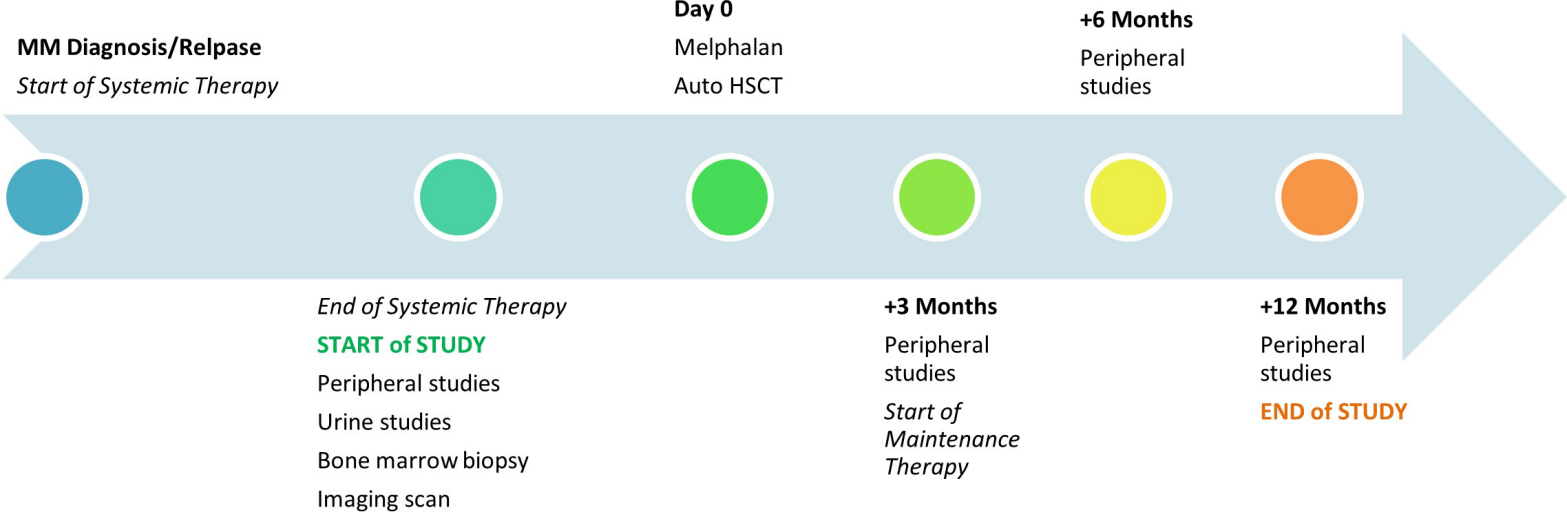
Study schema. All patients received systemic therapy (n= 44) for either newly diagnosed or R/R MM with the majority (n= 36) proceeding to Melphalan followed by autologous HSCT. Post-HSCT, maintenance therapy was started at +3 months. Studies performed pre-HSCT included serum sBCMA, standard serum and urine myeloma markers, BM biopsy, and an imaging scan. Studies obtained post-HSCT at +3, +6, +12 months (if timepoint reached by the patient by the end of the study) included serum sBCMA and standard serum myeloma markers.

Adult patients, age > 18 years, any sex and ethnicity, with newly diagnosed or R/R MM who underwent pre-autologous HSCT evaluation at our institution between 3/2023 and 12/2024 after receiving standard of care systemic therapy were included. Patients who received anti-BCMA therapy or investigational agents as part of their most recent systemic therapy, pregnant women, and staff or students at the institution were excluded. As part of this prospective observational correlative study, paired peripheral blood and BM samples were obtained at the start of the pre-HSCT evaluation, and for patients proceeding to HSCT, peripheral blood samples were obtained at pre-determined timepoints afterwards (**Figure 1**). Patients who did not proceed to HSCT were not followed beyond the pre-HSCT evaluation. Approval of the study was obtained from the Institutional Review Board at the University of Florida.

### Standard myeloma studies and response assessment

All patients underwent a pre-HSCT evaluation involving restaging of the MM. Serum standard myeloma markers were collected and included immunoglobulins (Ig), immunoglobulin free light chains (sFLC) [quantitation was performed on an Optilite analyzer (The Binding Site, San Diego, CA USA)], monoclonal spike (M-spike), and immunofixation (IFE). Urine M-spike and IFE were also obtained. Each patient’s type of Ig, either IgG or IgA, and type of sFLC, either kappa or lambda, was designated as their “involved” serum marker. Patients underwent a BM biopsy and aspirate. BM biopsies were evaluated by routine morphology and at least immunohistochemistry/*in-situ* hybridization for CD138 and kappa/lambda light chains. In addition to routine morphology, aspirates were also submitted for flow cytometric analysis including MRD assessment. BM overt disease was defined as malignant plasma cells identified on morphological/immunohistochemical analysis or by standard flow cytometry with a limit of detection (LOD) of 10^-1^. BM MRD was assessed by MRD flow cytometry with a sensitivity of 1 aberrant clonal plasma cell in 10^5^ nucleated cells following the EuroFlow methodology (7) and/or next-generation sequencing (NGS) with a LOD of 10^-6^.

To assess for extramedullary disease, patients had either a whole-body F-18-FDG positron emission tomography/computed tomography (PET/CT) or whole-body CT scan, as available. Despite being able to pick up new lesions, a limitation of the whole-body CT scan is that it cannot differentiate between treated and now non-active vs. continuously active myeloma lesions.

Pre-HSCT response to therapy was determined by the International Myeloma Working Group (IMWG) criteria: partial response (PR), very good partial response (VGPR), complete response (CR), stringent CR (sCR), or progression of disease (PD).

For patients who proceeded to autologous HSCT and reached these timepoints by the end of the study, serum standard myeloma markers were also obtained at +3, +6, and +12-months post-HSCT. Bone marrow biopsy, standard urine myeloma studies, and imaging studies were not repeated post-HSCT. High risk cytogenetics were defined as either t(4;14), t(14;16), deletion 17p, and/or 1q21 gain, on fluorescence *in situ* hybridization testing.

### Serum sBCMA detection by sandwich enzyme-linked immunosorbent assay (ELISA) testing

Serum sBCMA samples were obtained during the pre-HSCT evaluation and, if applicable, at the standard post-HSCT follow up timepoints (+3, +6, +12 months). To detect serum sBCMA, whole blood specimens were delivered on ice and either processed immediately or stored at 4°C for less than 24 hours (hr) (typically < 1 hr) before processing. Blood (1.0 mL) was transferred to micro sample tube serum gel (Sarstedt, Nümbrecht, Germany), centrifuged at 5,000 x g for 5 minutes, aliquoted into RNase, DNase free polypropylene microfuge tubes (Fisher Scientific), and immediately stored at -80°C until testing.

The DuoSet™ ELISA Development System Human BCMA/TNFRSF17 (R&D Systems Inc., Minneapolis, MN) and the DuoSet™ Ancillary Reagent Kit 2 were used to measure natural human BCMA in serum samples per manufacturer instructions (8). Briefly, kit reagents were prepared and stored as directed. Plates were sealed for incubation steps. The capture antibody was diluted in phosphate buffered saline (400 ng/mL), added to wells (100 uL), and incubated overnight at room temperature. Plates were washed 3X between each subsequent step as directed with 0.3 mL 1X PBS with 0.05% Tween 20 using automatic plate washer (Biotek 405|LS microplate washer; Winooski, VT). Samples and standards (31.2–2000 pg/mL) were diluted in reagent diluent and added to each well (100 uL) and incubated 2 hrs at room temperature. Detection antibody diluted in reagent diluent (200 ng/mL), added to wells (100 uL), and incubated 2 hrs at room temperature. Streptavidin-HRP (horseradish peroxidase) A was diluted 200-fold in reagent diluent, added to wells (100 uL), and incubated 20 min at room temperature in the dark. After the final wash step, 3,3’,5,5’-Tetramethylbenzidine (TMB) ELISA substrate solution was added to each well (100 uL), incubated 20 min at room temperature in the dark, and stopped with 2 N H_2_SO_4_ (50 uL). Optical density was determined immediately at 450 nm and wavelength corrected at 540 nm. For analysis, the average zero standard optical density was subtracted from each of the triplicate readings for standards, controls, and samples. The standard curve was created by generating a four-parameter logistic curve-fit (www.aatbio.com). A standard percent recovery between 80 – 120% was considered acceptable. For diluted samples, the concentration extrapolated from the standard curve was multiplied by the dilution factor. Average, standard deviation, and % coefficient of variation were then calculated.

### Statistical analysis

Summary statistics for baseline variables, stratified by transplantation status, were provided in a table format. Means, standard deviations (SD), medians, interquartile ranges (IQR), minimum and maximum were calculated for continuous variables. Counts and percentages were used for categorical variables. We conducted complete case analysis for each set of investigations by excluding observations with unavailable values for the variables in each analysis. Serum sBCMA at initial assessment pre-HSCT was not normally distributed from Anderson-Darling Test (p = 0.02). Thus, a nonparametric test, Wilcoxon rank sum test, was used to assess the difference of serum sBCMA between disease status groups, including BM overt disease vs. no overt disease, BM detectable MRD or no detectable MRD (MRD only/no MRD) vs. overt disease, out of normal range vs. normal range standard biomarkers, and positive vs. negative imaging scans. We also applied the Wilcoxon rank sum test as an exploratory analysis to examine the association between serum sBCMA and the standard serum and urine myeloma markers (within vs. out of normal range).

To account for potential confounding factors, we used multivariable logistic regressions to model the association between residual disease status (residual disease/no residual disease) and serum sBCMA, adjusting for covariates including demographic variables (age, race and sex) and potential confounding biomarkers (sFLC, serum kappa/lambda light chain ratio, Ig, serum M-spike, urine M-spike). Variance inflation factor was leveraged to examine the potential multicollinearity issues, from which we did not find any evidence of multicollinearity. The backward selection method based on Akaike information criterion was implemented to identify the most informative sets of variables. For an outcome variable of BM overt disease, we selected serum sBCMA and serum M-spike as exposure variables. For an outcome variable of BM MRD only/No MRD, we selected serum sBCMA, serum M-spike, and race as exposure variables.

In addition, we explored the potential application of baseline sBCMA as a predictor of disease status in the BM (overt or MRD only/No MRD). For interpretability, our prediction model for BM overt disease was developed using univariable logistic regression, with BM overt disease regressed on serum sBCMA. Given the limited sample size, we used the same dataset for both training and validation of the model and obtained an area under the receiver operating characteristic (AUROC) to assess model performance. Liu’s method (9) was used to optimize the combination of sensitivity and specificity. The prediction model for BM MRD only/No MRD was constructed in a similar manner.

Finally, descriptive analysis was performed for biomarkers at pre-HSCT and different follow-up times post-HSCT (+3, +6, and +12 months). For this part of the data with repeated measurement, we leveraged the linear mixed effects models (LMM) to analyze serum sBCMA over time against each biomarker. The LMM includes both fixed effects of different biomarkers on serum sBCMA and random effects of individual variability among subjects.

A p-value < 0.05 was defined as statistically significant. R Statistical Software (v4.1.2; R Core Team 2021) was used to perform all analyses.

## Results

### Patient population

Altogether, 52 patients were screened for the study with 44 enrolled. Of these, 36 proceeded to autologous HSCT with Melphalan conditioning and 8 did not. Their characteristics, type of systemic therapy, and pre-HSCT restaging studies are presented in **Table 1**.

**Table 1:** Patient demographics, diagnosis, systemic therapy, and myeloma studies pre-HSCT.

Characteristic	Transplanted patients n= 36 (%)	Non-transplanted patients n= 8 (%)
Median Age, range (years)	65.5 (41-78)	64.5 (57-70)
Sex, male	24 (66.7)	3 (37.5)
Ethnicity		
Non-Hispanic or Latino	34 (94.4)	6 (75)
Hispanic or Latino	2 (5.6)	2 (25)
Race		
White	27 (75)	5 (71.4)
Black	9 (25)	2 (28.6)
Type of MM		
IgG kappa	16 (44.4)	1 (12.5)
IgG lambda	4 (11.1)	2 (25)
IgA kappa	5 (13.9)	1 (12.5)
IgA lambda	4 (11.1)	0
IgD lambda	0	1 (12.5)
Kappa light chain	5 (13.9)	3 (37.5)
Lambda light chain	2 (5.6)	0
Stage of MM		
Durie-Salmon Staging, IIA-IIIB		0
I	2 (5.6)	
II	2 (5.6)	
III	6 (16.6)	
Revised or International Staging System		
I	4 (11.1)	3 (37.5)
II	9 (25)	1 (12.5)
III	2 (5.6)	2 (25)
N/A	11 (30.5)	2 (25)
High Risk Cytogenetics, present	10 (27.8)	3 (37.5)
Systemic Therapy		
Triplet regimen (lenalidomide/bortezomib/dexamethasone)	18 (50)	1 (12.5)
Quadruplet regimen (daratumumab-lenalidomide/bortezomib/dexamethasone)	13 (36.1)	2 (25)
Other triplet regimens	5 (13.9)	1 (12.5)
Required 2 lines of therapy prior to HSCT	4 (11.1)	4 (50)
Received supplemental radiation therapy	5 (13.9)	0
Response to Therapy Pre-HSCT		
PR	14 (38.9)	2 (25)
VGPR	15 (41.7)	2 (25)
CR	3 (8.3)	1 (12.5)
sCR	4 (11.1)	2 (25)
N/A	0	1 (12.5)
Biomarkers Pre-HSCT		
Serum sBCMA, ng/mL		
Mean (SD)	18.1 (11.7)	9.7 (5.2)
Median (Q1 -Q3)	15.4 (9.0-26.3)	10.3 (5.5-13.9)
Range	2.0-50.3	2.0-16.6
Involved Ig, mg/dL		
Mean (SD)	548.7 (292.6)	340.3 (368.3)
Median (Q1-Q3)	490.0 (365.0-773.0)	311.0 (16.0-467.0)
Range	25.0-1069.0	9.5-898.0
N/A	7	3
Involved sFLC, mg/dL		
Mean (SD)	2.8 (3.0)	2.6 (4.1)
Median (Q1-Q3)	1.6 (0.7-4.1)	0.9 (0.7-2.4)
Range	0.3-12.5	0.3-12.5
Kappa/lambda LC ratio		
Mean (SD)	2.5 (2.2)	1.6 (0.9)
Median (Q1-Q3)	1.8 (1.3-2.9)	1.6 (1.1-2.2)
Range	0.1-10.4	0.1-3.0
N/A	1	0
Serum M-spike, g/dL		
Mean (SD)	0.3 (1.1)	0.1 (0.2)
Median (Q1-Q3)	0.1 (0.0-0.2)	0 (0.0-0.1)
Range	0.0-6.9	0-0.73
Serum IFE		
Detectable, n (%)	25 (69.4)	2 (25)
Undetectable, n (%)	11 (30.6)	6 (75)
Urine M-spike, %		0
Mean (SD)	2.6 (7.8)	
Median (Q1-Q3)	0.0 (0.0-0.0)	
Range	0.0-37.0	
N/A	1	3
Urine IFE		
Detectable, n (%)	16	1
Undetectable, n (%)	19	4
N/A	1	3
Extramedullary Disease per Imaging		
FDG-PET/CT Scan		
Active (FDG avid) MM Lesions	5	0
Non-Active MM Lesions	15	3
CT Scan		
MM Lesions Present	12	3
MM Lesions not Present	4	1
N/A	0	1
BM Aspirate		
Overt Disease, n (%)	12 (33.3)	1 (12.5)
Percent MM in the BM		
Mean (SD)	4.1 (16.7)	0.4 (1.1)
Median (Q1-Q3)	0.0 (0.0-5.0)	0.0 (0.0-0.0)
Range	0.0-100.0	0.0-3.0
Detectable MRD, n (%)	28 (77.8)	4 (50)
Both MRD Flow Cytometry and NGS, n	1	1
MRD Flow Cytometry, n	14	1
NGS, n	9	2
Standard Flow Cytometry, n	4	0
Undetectable MRD, n (%)	4 (11.1)	2 (25)
MRD Flow Cytometry, n	4	2
NGS, n	0	0
MRD N/A, n (%)	4 (11.1)	2 (25)
MRD only, n (%)	16 (44.4)	3 (37.5)
MRD only/No MRD, n (%)	20 (55.6)	5 (62.5)
Type of Maintenance Therapy Post-HSCT, Transplanted patients only		
lenalidomide daratumumab plus lenalidomide	16 (44.4) 6 (16.7)	
bortezomib	5 (13.9)	
daratumumab	4 (11.1)	
pomalidomide	1 (2.8)	
N/A	4 (11.1)	

N/A, not available or applicable; SD, standard deviation; Q, quartile. Normal values: IgG (635-1616) mg/dL; IgA (70-433) mg/dL; KLC (0.33-1.94) mg/dL; LLC (0.57-2.63) mg/dL; serum M-spike 0 g/dL; serum IFE undetectable; urine M-spike 0%; urine IFE undetectable.

Of note, the serum sBCMA (15.98 ng/mL) for one patient was drawn at the initial pre-HSCT evaluation only when the patient was found to have refractory to first line therapy MM with 100% malignant plasma cells in the BM and was not repeated following salvage systemic therapy and prior to proceeding to HSCT.

Regarding BM disease burden, in all cases with BM overt disease, patients also had BM MRD. Thus, in a few cases (n= 4) where BM MRD test results were unavailable but disease was detectable by standard flow cytometry, both BM overt disease and MRD were marked as present.

Patients who did not proceed to HSCT either had a contraindication (n= 5) or declined HSCT (n= 3). **Table 2** lists the post-HSCT peripheral myeloma studies of the transplanted patients. By +1 year 6 months post-HSCT, 2 patients had experienced progression of disease, and 2 patients were deceased (1 due to progression of disease and 1 due to myocardial infarction).

**Table 2:** Myeloma studies in the transplanted patients at predetermined timepoints post-HSCT.

Peripheral Biomarkers Post-HSCT	+3 Months	+6 Months	+12 Months
Serum sBCMA, ng/mL			
Mean (SD)	12.7 (11.3)	12.8 (9.0)	14.0 (9.1)
Median (Q1 -Q3)	10.8 (4.8-16.1)	10.8 (6.0-18.1)	11.4 (7.2-18.4)
Range	1.1-47.9	0.9-34.0	3.5-33.6
N/A	5	11	22
Involved Ig, mg/dL			
Mean (SD)	471.9 (371.3)	536.5 (328.4)	680.4 (295.6)
Median (Q1-Q3)	422.0 (237.0-605.0)	490.0 (320.0-688.0)	639.0 (422.0-903.0)
Range	17.0-1526.0	21.0-1243.0	274.0-1249.0
N/A	11	17	25
Involved sFLC, mg/dL			
Mean (SD)	1.7 (1.7)	2.1 (1.8)	2.2 (2.0)
Median (Q1-Q3)	1.0 (0.4-2.5)	1.4 (0.6-3.2)	1.6 (0.9-3.3)
Range	0.1-6.4	0.2-6.9	0.2-6.9
N/A	5	12	22
Kappa/lambda LC ratio			
Mean (SD)	2.7 (3.3)	2.2 (1.7)	2.0 (1.6)
Median (Q1-Q3)	1.7 (1.4-2.5)	1.5 (1.2-2.4)	1.6 (1.3-1.9)
Range	0.6-15.0	0.3-7.0	0.8-7.2
N/A	5	13	22
Serum M-spike, g/dL			
Mean (SD)	0.1 (0.2)	0.1 (0.2)	0.1 (0.3)
Median (Q1-Q3)	0.0 (0.0-0.1)	0.0 (0.0-0.1)	0.0 (0.0-0.0)
Range	0.0-1.0	0.0-0.8	0.0-0.9
N/A	5	12	22
Serum IFE			
Detectable, n (%)	17 (54.8)	13 (54.2)	9 (64.3)
Undetectable, n (%)	14 (45.2)	11 (45.8)	5 (35.7)
N/A	5	12	22

N/A, not available or applicable; SD, standard deviation; Q, quartile. Normal values: IgG (635-1616) mg/dL; IgA (70-433) mg/dL; KLC (0.33-1.94) mg/dL; LLC (0.57-2.63) mg/dL; serum M-spike 0 g/dL; serum IFE undetectable.

### Relationship between serum sBCMA and MM tumor burden

**Figure 2:**
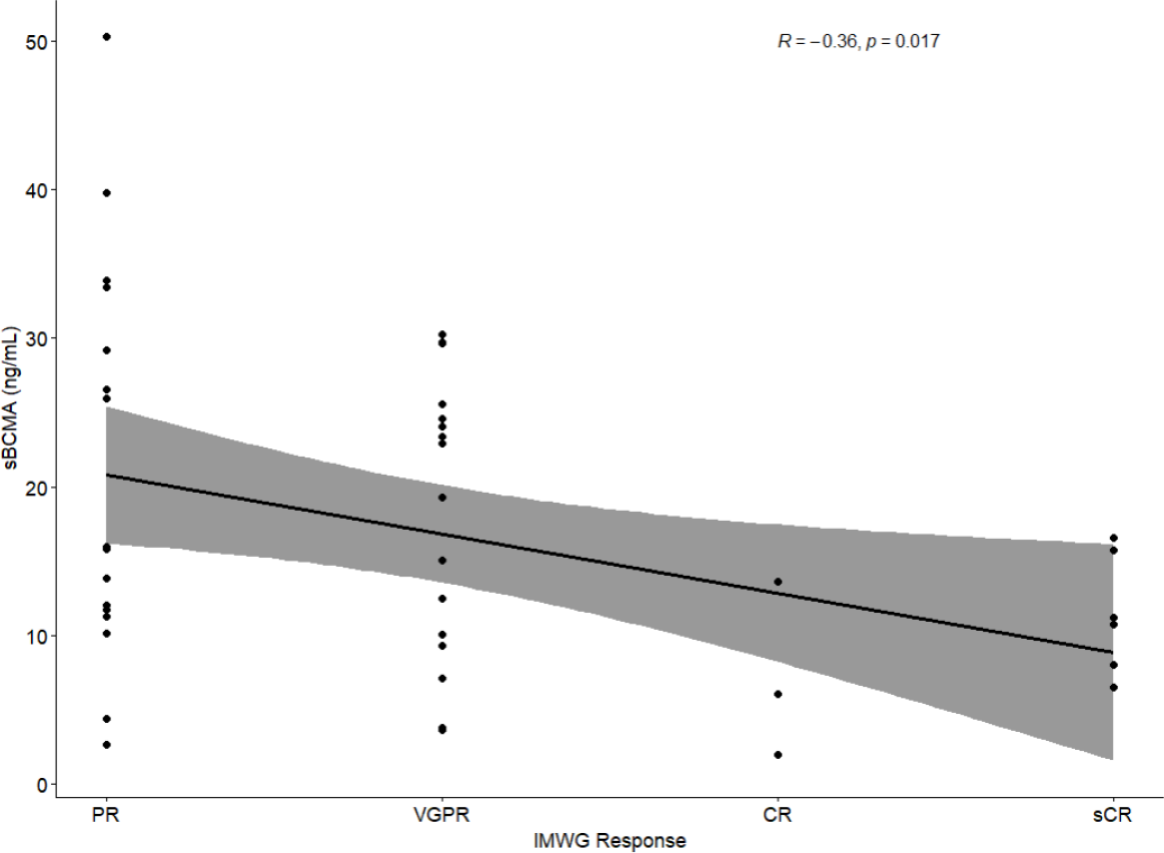
Association of serum sBCMA with tumor burden by IMWG response category. Lower levels of serum sBCMA associated with better responses to systemic therapy.

For patients staged by IMWG criteria pre-HSCT, higher levels of serum sBCMA was associated with higher levels of tumor burden by IMWG response category (correlation coefficient (r)= -0.36, p= 0.01) (**Figure 2**).

**Figure 3:**
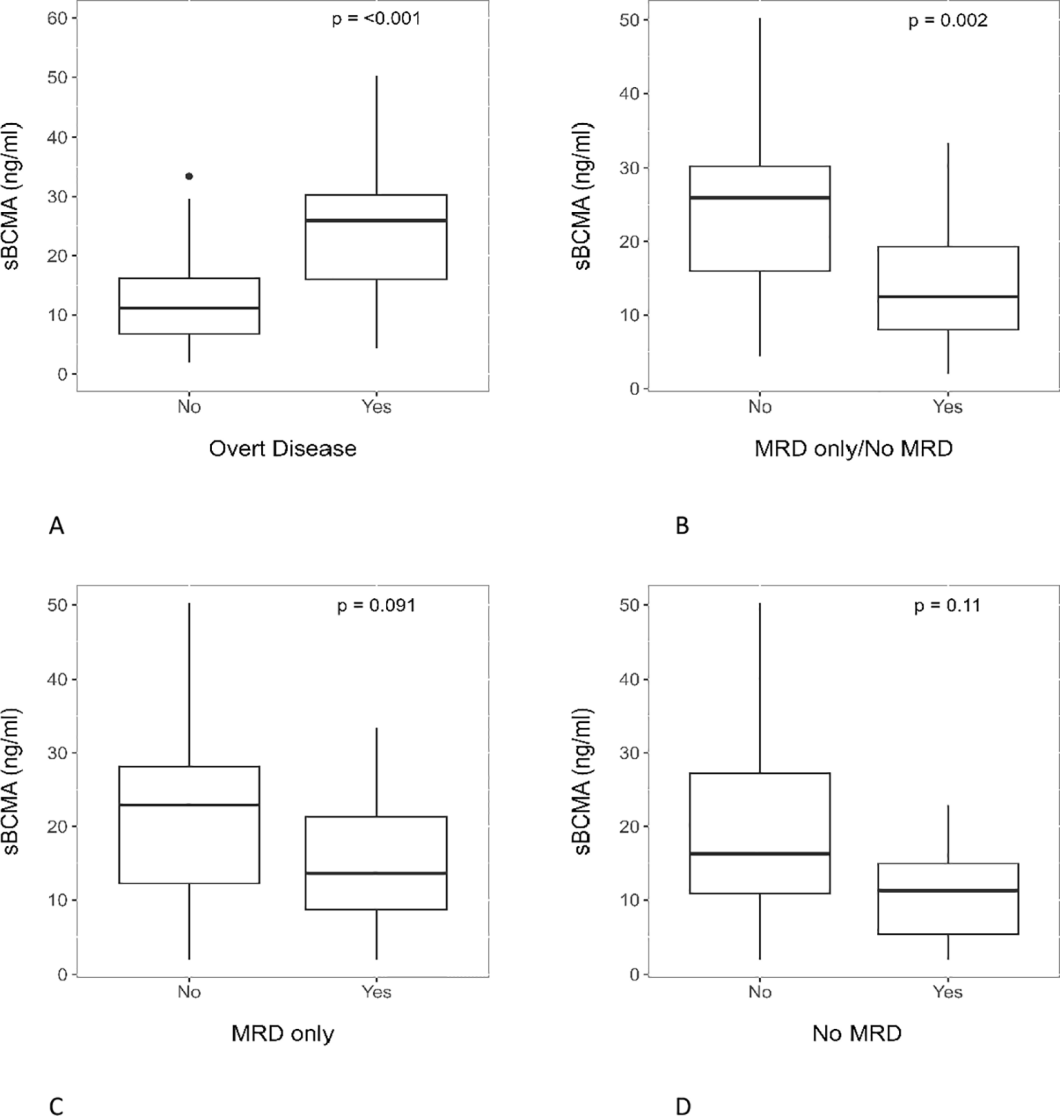
Serum sBCMA association with BM MM burden. Serum sBCMA was significantly associated with both **(A)** BM overt disease and **(B)** BM MRD only/No MRD. However, it was not associated with **(C)** BM MRD only or **(D)** No MRD.

Focusing on the BM, we analyzed the relationship between the myeloma burden within the compartment and corresponding peripheral sBCMA. All patients were categorized into BM overt disease vs. no overt disease (n vs. n) (13 vs. 31). Overt disease in the BM associated significantly with higher serum sBCMA (p < 0.001) (**Figure 3A**). Excluding patients with no BM overt disease but unavailable MRD (n= 6), patients were also categorized into BM overt disease vs. MRD only/No MRD (13 vs. 25). BM MRD only/No MRD associated significantly with lower serum sBCMA (p= 0.002) (**Figure 3B**). Due to the relatively small number of patients in the study, by itself, neither BM MRD only (p= 0.09) (**Figure 3C**) nor No MRD (p= 0.11) (**Figure 3D**) associated significantly with serum sBCMA.

Next, we examined the associations of serum sBCMA with standard serum or urine myeloma markers pre-HSCT and up to +12 months post-HSCT where applicable. All patients were grouped according to out of vs. within normal range standard myeloma markers: IgG (13 vs. 10), IgA (6 vs. 4), kappa LC (13 vs. 18), lambda LC (9 vs. 4), kappa/lambda LC ratio (28 vs. 15), serum M-spike (24 vs. 20), serum IFE (17 vs. 27), urine M-spike (35 vs. 5), and urine IFE (23 vs. 17). Involved Ig and involved sFLC were evaluated as continuous variables. Applying the Wilcoxon rank sum test as an exploratory analysis to examine the association between pre-HSCT serum sBCMA and standard myeloma markers revealed a significant association of serum sBCMA with involved sFLC (p < 0.001), kappa LC (p < 0.001), kappa/lambda LC ratio (p = 0.03), and serum IFE (p = 0.04). On the other hand, there was no significant association of serum sBCMA with lambda LC (p= 0.5), involved Ig (p= 0.2), IgG (p= 0.3), IgA (p= 0.6), serum M-spike (p= 0.06), urine M-spike (p= 0.06), or urine IFE (p= 0.4).

**Figure 4:**
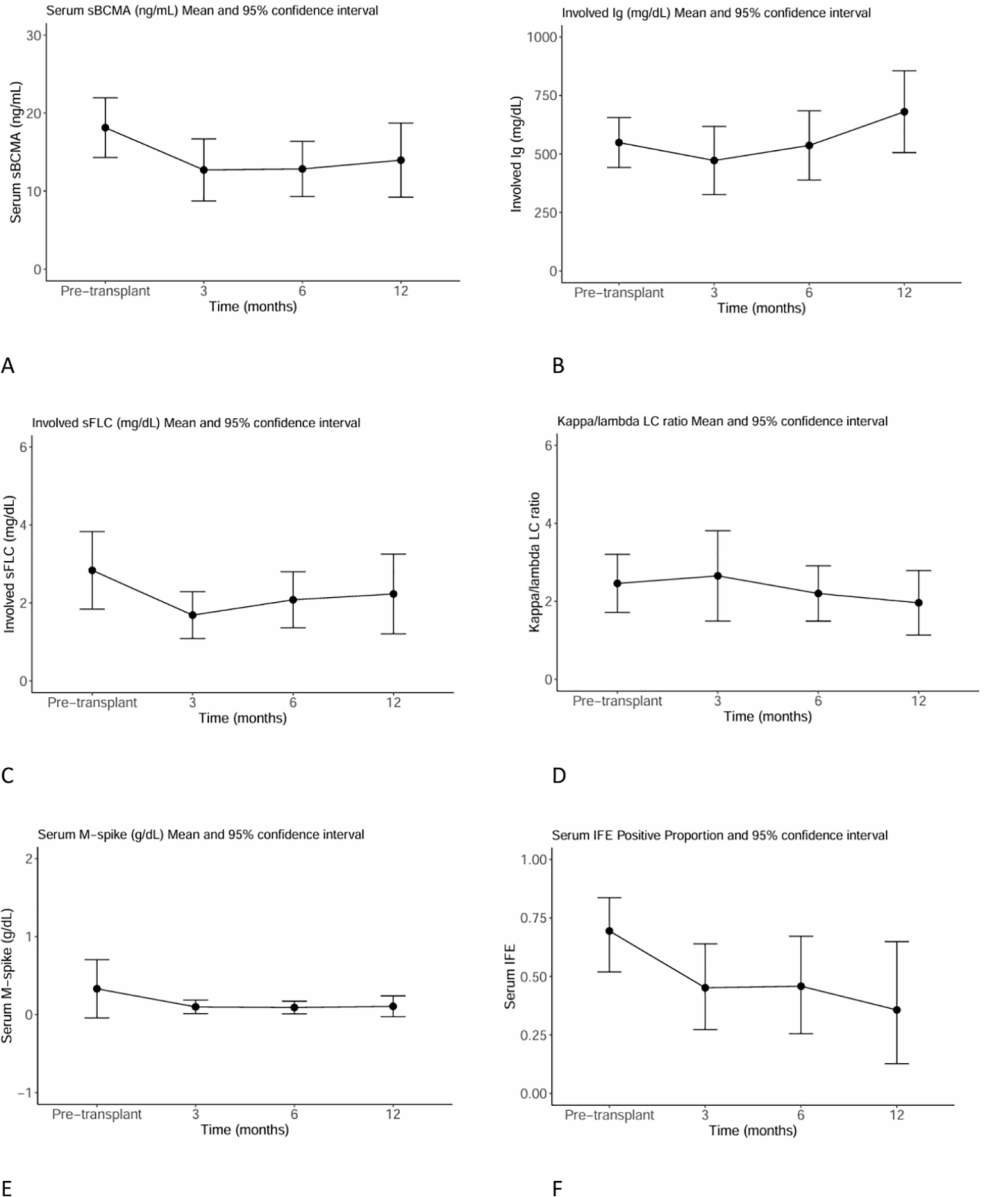
Levels of serum sBCMA and standard myeloma markers for transplant recipients (n=36) pre-HSCT and at +3, +6, and +12 months post-HSCT. **(A)** Serum sBCMA **(B)** Involved Ig **(C)** Involved sFLC **(D)** Kappa/lambda LC ratio **(E)** Serum M-spike **(F)** Serum IFE.

Levels of serum sBCMA and standard serum myeloma markers for transplant recipients pre- and post-HSCT are graphed in **Figure 4**. By integrating data from pre-HSCT, +3, +6, and +12 months post-HSCT, we detected significant associations between sBCMA with involved Ig (p = 0.02), involved sFLC (p < 0.001), kappa/lambda LC ratio (p = 0.008), and serum IFE (p = 0.003). Serum sBCMA was again not found to be significantly associated with serum M-spike (p=0.3).

With respect to extramedullary disease on imaging, patients were marked as having either myeloma lesions present on CT scan vs. no lesions (15 vs. 5) or active vs. non-active FDG-avid lesions on PET/CT scan (5 vs. 18). Imaging was unavailable for one patient. Extramedullary disease was not significantly associated with serum sBCMA (p= 0.2).

### Multivariable logistic regressions

We performed multivariable logistic regressions to model the association between pre-HSCT BM residual disease status and serum sBCMA. Higher value of serum sBCMA indicated higher odds of overt disease in the BM (odds ratio (OR) = 1.12, p = 0.007)and lower odds of MRD only/No MRD (OR = 0.91, p = 0.03).

### Predictive modeling

**Figure 5:**
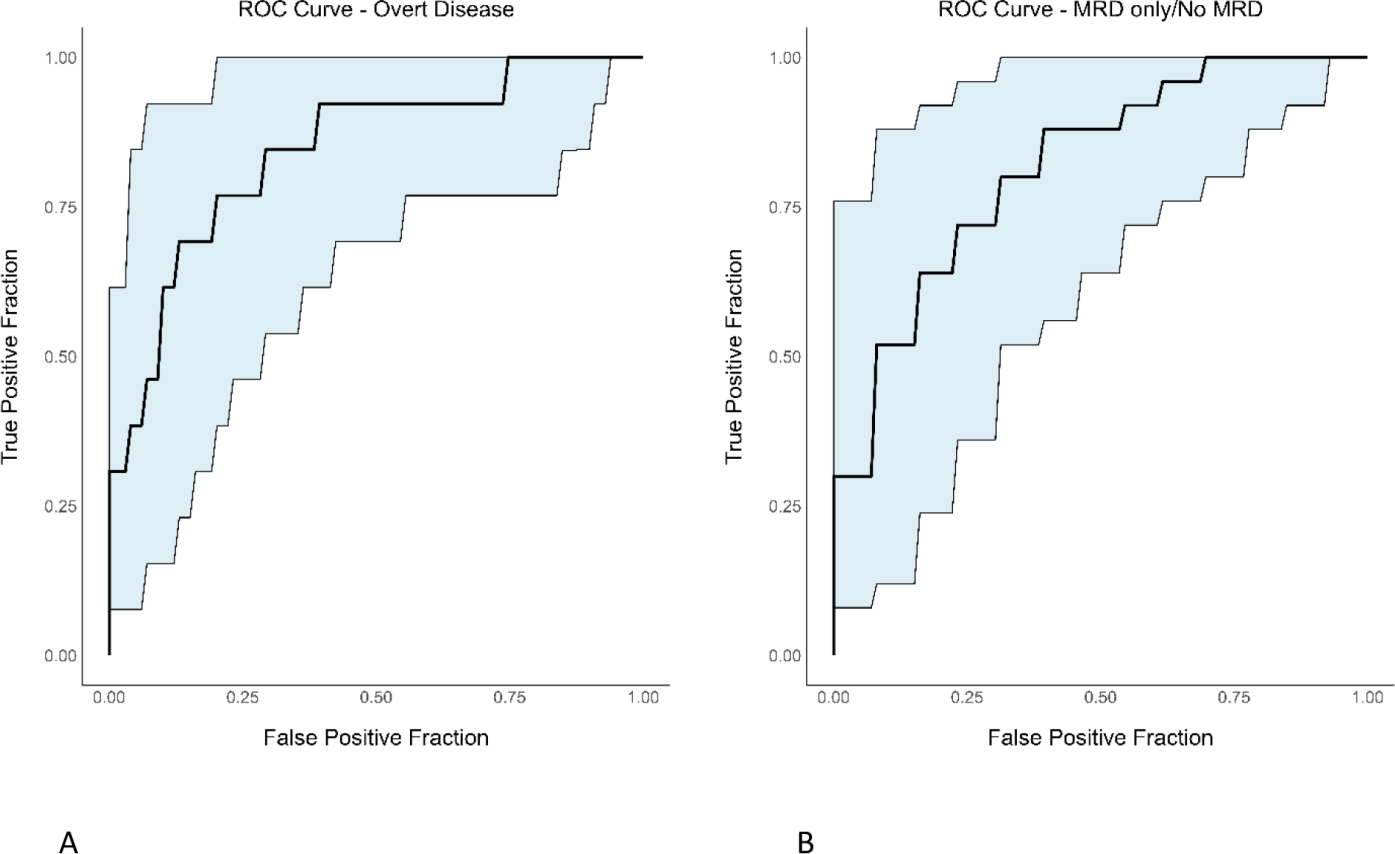
ROC curves with confidence intervals evaluating the performance of prediction models for BM overt disease and MRD only/No MRD using serum sBCMA. **(A)** BM overt disease **(B)** BM MRD only/No MRD.

Finally, using univariable logistic regression, we wanted to examine pre-HSCT serum sBCMA as a predictor for pre-HSCT BM disease status. For BM overt disease prediction, we calculated an AUROC of 0.83 to assess model performance (**Figure 5A**). Optimal sensitivity (or true positive fraction) was 76.92% and specificity (or 1-false positive fraction) was 74.19%. This method resulted in a positive predictive value of 56%, a negative predictive value of 88%, and an overall accuracy of 75%. For BM MRD only/No MRD disease prediction, we found AUROC of 0.81; optimal sensitivity and specificity of 84% and 69.23%, respectively (**Figure 5B**). The positive predictive value was 84% while the negative predictive value was 69% with an overall accuracy of 79%.

## Discussion

In this prospective observational correlative study, we found that in patients with MM pre-HSCT, lower serum sBCMA associated significantly with absent overt disease and with MRD only/No MRD in the BM, as well as with the standard serum myeloma markers involved sFLC, kappa LC, kappa/lambda LC ratio, and serum IFE.

We examined serum sBCMA as a potential marker of MRD as it is a ubiquitous protein present in the peripheral blood of patients with MM (6), measuring it in a low disease burden state such as post systemic therapy/pre-HSCT and further post-HSCT and during maintenance therapy. Even post treatment, all patients in the study had a quantifiable serum sBCMA at each evaluable timepoint. The mean serum sBCMA level at pre-HSCT was consistent with previously reported levels of patients in CR (20 ng/mL) and none in PD (750 ng/mL) (10). For patients proceeding to HSCT, the mean level decreased further afterwards, and up to +12 months post-HSCT, remained lower compared to pre-HSCT. This trend was anticipated based on a study demonstrating the quantification and temporal decrease of serum sBCMA to various therapies, including belantamab mafodotin and teclistamab, in both newly diagnosed and R/R MM (11). In contrast to our results, following administration of the anti-BCMA chimeric antigen receptor therapy, idecabtagene vicleucel, serum sBCMA nadired within 3 months and became undetectable in 63% of patients in PR, 81% in VGPR, and 95% in CR/sCR (12). Given anti-BCMA therapies were excluded and none of the patients received cellular immunotherapy in the current study, this suggests that the type of therapy impacts the degree of serum sBCMA level decrease.

Elevation of serum sBCMA has been shown to predict relapse of MM (13). Unfortunately, the study population here was too small, and the overall follow-up time and intervals used were inadequate, to determine how well it can serve to detect early relapses. For the two patients in this study who experienced progression of disease, one at +1 year post-HSCT, the latest available serum sBCMA at +6 months had increased by only 3 units compared to pre-HSCT; and for the other patient at +1 year 6 months post-HSCT, the latest available serum sBCMA at +1 year was stable compared to pre-HSCT. Due to patients being off site, serum sBCMA levels were not collected at the time of disease progression to observe any potential change in trends.

We used pre-HSCT serum sBCMA levels to determine the marker’s relationship with total MM burden and by compartment. In agreement with published works (6, 13, 14), we found an association of higher levels of serum sBCMA with high disease burden as determined comprehensively by IMWG response category. Similarly to other studies (13, 14), overt disease in the BM associated significantly with serum sBCMA. However, we additionally found that in the absence of overt disease in the BM, there was a significant association between serum sBCMA and BM MRD only/No MRD. Our findings regarding the relationship between serum sBCMA and both overt disease and MRD only/No MRD in the BM were confirmed on multivariable logistic regression modeling.

Prior studies on serum sBCMA as a marker of MRD are limited. Serum sBCMA has been shown to correlate with undetectable MRD by NGS only in the periphery of patients with MM undergoing a trial with a bispecific antibody (15). Here, we did not obtain NGS peripherally, but we did find a significant association of pre-HSCT serum sBCMA with standard serum myeloma markers, including involved sFLC, kappa LC, kappa/lambda LC ratio, and serum IFE. In the subgroup of transplant recipients, serum sBCMA showed a significant association with involved sFLC, kappa/lambda LC ratio, serum IFE, and with involved Ig, longitudinally, pre-HSCT and up to +12 months post-HSCT. While our results were concurrent with another study that illustrated a strong association between serum sBCMA with involved sFLC during the course of disease (13), we did not find any relationship with serum M-spike perhaps due to our low mean serum M-spike value.

We also did not find that pre-HSCT serum sBCMA associated with urine myeloma markers. In a high tumor burden state in patients with R/R MM undergoing treatment with the anti-BCMA/CD3 bispecific antibody, linvoseltamab, serum sBCMA has been shown to correlate modestly with urine protein (16). Evidence is lacking in low tumor burden states. We did not measure sBCMA in the urine, but there is also insufficient knowledge on whether serum sBCMA can be secreted in the urine and at what rate. It has been reported, however, that the concentration of serum sBCMA is independent of renal function (11), which increases its value as a potential biomarker in MM, where patients may have renal insufficiency.

In this study, there was no association of serum sBCMA with presence of extramedullary disease on imaging. As demonstrated in cases of a solitary plasmacytoma, the protein can be elevated due to extramedullary disease only (11). A decrease in standardized uptake value of lesions on PET scans has been shown to correlate with a decrease in sBCMA levels, and negative PET scans with a stable, low detectable serum sBCMA (13). These findings imply serum sBCMA is elevated with active myeloma lesions on imaging. In our study, due to the unavailability of PET/CT scans for all patients, we were not able to identify all radiologically active extramedullary disease as a potential peripheral source of serum sBCMA, which could have affected results.

We explored the value of serum sBCMA as a predictor of BM myeloma burden pre-HSCT. The protein has already been shown to be predictive of progression free and overall survival in patients with MM undergoing first line or salvage therapy (13). Here, we found that while serum sBCMA had moderate likelihood of predicting overt BM disease, it had high likelihood of predicting MRD only/No MRD BM disease. To our knowledge, this is the first study evaluating serum sBCMA as a potential non-invasive, peripheral biomarker of MRD pre- and post-autologous HSCT for MM.

Biomarker development is ongoing in the field of MM. To facilitate diagnosis, prognosis, as well as therapy selection, escalation, and de-escalation, different types of biomarkers, including clinical, imaging, serological, genetic, and protein, are evaluated individually and in combination (17). For example, signaling lymphocytic activation molecule F2 (SLAMF7), has been studied as a biomarker for diagnostic, prognostic, and therapeutic purposes (17, 18). In a similar way to BCMA, SLAMF7 can be surface-bound or cleaved off plasma cells to become soluble in the serum (19). Elevations in SLAMF7 have been associated with aggressive MM expressing t(4;14), and the marker has been used to target the disease with the monoclonal antibody elotuzumab, currently approved for R/R MM (17). Additionally, about 30% of patients with MM harbor mutations in the mitogen-activated protein kinase (MAPK) pathway, which includes the *KRAS*, *NRAS* and *BRAF* genes, making it the most affected signaling pathway and, therefore, an appealing target for biomarker exploration (17). In the BRAF-RAS pathway, the *CIC* mutation has been identified as a potential biomarker, associated with extramedullary dissemination of MM and poor prognosis (20). Some other examples of novel biomarkers include miRNAs, non-coding RNA and splicing events (17). Discovering new biomarkers, and if appropriate, assessing them for MRD detection is crucial for effective management of patients with MM.

Strengths of this study include the prospective collection of samples and the detection of serum sBCMA by a commercial, widely available assay. Limitations are the single center setting of the study with a relatively low number of patients limiting its statistical power, especially in the subgroup analyses, and an unavailable separate patient cohort for independent validation of the results. The variations in sBCMA levels observed here, with some patients in a better IMWG response category having higher sBCMA values than others in a worse IMWG category and vice versa, can also be elucidated through further data collection in a larger patient cohort. Another limitation of the study is the lack of BM MRD data for all patients and lack of BM MRD assessment by NGS for the patients who had their MRD tested due to either an MRD sample not being collected, lack of medical insurance coverage for MRD testing, or polyclonal results on the diagnostic NGS sample. When MRD testing was preformed, the use of different methods led to MRD detection at different depths.

In conclusion, in patients with MM, pre-HSCT serum sBCMA associated not only with BM overt disease, but also with BM detectable and/or below LOD MRD. It was also associated with standard serum myeloma markers, including involved sFLC, kappa LC, kappa/lambda LC ratio, and serum IFE. Further studies of serum sBCMA are warranted to evaluate it as a potential non-invasive biomarker of MRD in patients with MM.

## Data Availability

The raw data supporting the conclusions of this article will be made available by the authors, without undue reservation.
